# Information Relative to the Investigation of the Influence of Climate on Health

**Published:** 1895-06

**Authors:** Mark W. Harrington

**Affiliations:** Chief of Bureau


### INFORMATION RELATIVE TO THE INVESTIGATION OF THE INFLUENCE OF CLIMATE ON HEALTH.

CIRCULAR NO. 4. SANITARY CLIMATOLOGY. U. S. DEPARTMENT OF AGRICULTURE, WEATHER BUREAU, WASHINGTON, D. C., MARCH 23D, 1895.

The investigation of the influence of climate on health that has been undertaken by the Weather Bureau was first made public by the issuing of a circular, which was published in THE HOMŒOPATHIC PHYSICIAN for February, 1895, page 82.

The methods and details to be pursued in gathering together the data required for the investigation had not been matured when the circular was published, and its purpose was mainly to elicit suggestions and to gain some tangible estimate of the extent of the statistics, and of the number of co-operators that could probably be expected. At the same time, letters were addressed to the various officials of this Bureau, scattered throughout the country, asking them to ascertain the methods of recording and reporting vital statistics in vogue in their respective localities. From the information obtained by these means, the plan and details of the work have been determined so far as the possibilities of an untried experiment will permit.

The investigation of the relation and application of climatic agencies to hygienic conditions requires the collection and collation of the essential facts of both meteorology and hygiene. While the Bureau will obtain the necessary climatic data from the records of its various meteorologic stations, it must look to the voluntary assistance of the medical and sanitary professions for the statistics of mortality and morbidity; and the ultimate value of the investigation will depend in a great degree upon the interest these professions take in the enterprise.

### VITAL STATISTICS.

Wherever it is practicable, the mortality and morbidity statistics will be collected by weekly periods.

The kind of facts that are desired relative to mortality and the shape in which they are asked to be returned, will appear from the blank form prepared for the purpose, of which copies may be obtaiaed from the department.

The form is designed for the use of boards of health and health officers, in returning the deaths occurring in their respective municipalities, districts, and States during any calendar week. Provision is also made thereon for reporting such diseases as may be registered as dangerous to public health.

Co-operators will be supplied with penalty envelopes to be used in corresponding with the Bureau upon official matters, as in asking for supplies or information necessary to their assistance in the investigation; and co-operators are requested to anticipate by timely notification the exhaustion of their supplies.

The Bureau requests that co-operators will forward their reports as soon after the end of the week as convenient, to the end that the statistics may be received in time to afford ample opportunity for compilation and publication while the events are still fresh enough to be recalled by either the general reader or the student of medical climatology.

### COMPILATION.

The vital and meteorologic statistics, having been received, will be collated by general averages and by particular and selected events, as the comparison of the general mortality with the average conditions of the weather for the week, and the passage of storms and cold or hot waves, the appearance of epidemics, etc. Also, in instances as well-defined weather disturbances, comparisons of vital and meteorologic statistics will be made by daily periods. For example, a storm appearing in the western part of the country will be followed day by day as it passes eastward across the country, and the illness and deaths reported for these days from the localities traversed will be compiled and compared with the same kind of facts reported both before and after the storm. The same plan of treatment will be pursued in dealing with hot and cold waves.

By these methods we may hope to be able to give, in time, definite information as to how much and how the accidental and constant variations of the weather affect the sick and well, and in what way the present forecasts and weather charts can be used in both curative and preventive medicine.

The calendar week has been adopted as the period of time for collecting statistics and making the general comparison; because in longer periods, for instance a month, the evidence of extreme fluctuations in either the meteorologic or sanitary conditions is more or less smoothed out in proportion to the length of time during which the events happened. Also, because it has the advantage over other short arbitrary periods in being familiar to all, and one by which so many of our ordinary events and actions are reckoned.

### THE PUBLICATION.

A publication containing the collected and compiled facts will be issued monthly. This publication will comprise, in the shape of tables, charts, and diagrams, the chief meteorologic factors as observed and recorded by the officials of the Weather Bureau, and the statistics of mortality and morbidity as reported by the various public health officials and by individual physicians; also brief statements of the general sanitary conditions of the different localities, especially as they may have been influenced by the weather.

Under no circumstances will discriminating or advisory notices of any locality be published, the entire aim of the Bureau being to collect the facts and statistics for the sanitary and medical profession, and for the general public, to use in such ways and for such purposes as they may see fit.

MARK W. HARRINGTON,
*Chief of Bureau.*

